# Impact of Esophageal Dilation and Smoking on Bronchoalveolar Lavage Immune Profiles, Cellular Distribution, and Lipid-Laden Macrophage Index in Idiopathic Pulmonary Fibrosis

**DOI:** 10.3390/jcm15051761

**Published:** 2026-02-26

**Authors:** Soner Demirbaş, Celalettin Korkmaz, Adil Zamani, Pembe Oltulu, Hülya Vatansev, Pınar Diydem Yılmaz, Şebnem Yosunkaya, Turgut Teke

**Affiliations:** 1 Department of Pulmonary Diseases, Faculty of Medicine, Necmettin Erbakan University, Konya 42080, Türkiye; sdemirbas@erbakan.edu.tr (S.D.); azamani@erbakan.edu.tr (A.Z.); hulyavatansev@erbakan.edu.tr (H.V.); syosunkaya@erbakan.edu.tr (Ş.Y.); tteke@erbakan.edu.tr (T.T.); 2 Department of Pathology, Faculty of Medicine, Necmettin Erbakan University, Konya 42080, Türkiye; poltulu@erbakan.edu.tr; 3 Department of Radiology, Faculty of Medicine, Necmettin Erbakan University, Konya 42080, Türkiye; pdyilmaz@erbakan.edu.tr

**Keywords:** idiopathic pulmonary fibrosis (IPF), gastroesophageal reflux disease (GERD), bronchoalveolar lavage (BAL), esophageal dilation, lipid-laden alveolar macrophage index (LLMI), CD4+ T-lymphocytes

## Abstract

**Background:** Idiopathic pulmonary fibrosis (IPF) is a progressive interstitial lung disease with a poor prognosis. Esophageal dilation and hiatal hernia are common in IPF and may facilitate microaspiration, exacerbating inflammation. We investigated the relationship between radiological esophageal dilation, smoking status, and bronchoalveolar lavage (BAL) cellular and immunological profiles in IPF patients. **Methods:** This retrospective study included 71 IPF patients. Esophageal diameters were measured at four levels (L1–L4) via high-resolution computed tomography (HRCT). BAL fluid was analyzed for differential cell counts, the Lipid-Laden Macrophage Index (LLMI), and T-lymphocyte subsets (CD4+, CD8+) using flow cytometry. **Results:** Esophageal dilation (diameter >10 mm) was present in 52.1% of patients, and 36.6% had a hiatal hernia. A significant negative correlation was found between distal esophageal dilation (L4) and BAL CD4+ counts (r = −0.267, *p* = 0.024). Similarly, the mean maximum esophageal diameter negatively correlated with BAL CD4+ levels (r = −0.288, *p* = 0.015). Patients with hiatal hernia had significantly higher BAL neutrophil percentages than those without (20.0% ± 4.39% vs. 8.93% ± 2.0%, *p* = 0.047). Furthermore, smokers exhibited significantly lower BAL CD4+ levels than non-smokers (*p* = 0.042). No significant correlation was found between esophageal dilation and the LLMI (*p* > 0.05). **Conclusions:** Esophageal dilation is significantly associated with altered local immune profiles in IPF. The negative correlation between distal esophageal dilation and BAL CD4+ counts, plus the link between hiatal hernia and neutrophilic inflammation, suggests an interplay between esophageal dysfunction and the pulmonary immune microenvironment. Radiological assessment of esophageal dilation may serve as a non-invasive surrogate marker for identifying high-risk clinical phenotypes in IPF.

## 1. Introduction

Idiopathic pulmonary fibrosis (IPF) is a chronic, fibrosing interstitial pneumonia of unknown etiology, characterized by the radiologic and histologic patterns of usual interstitial pneumonia (UIP). Typically affecting older adults, IPF is distinguished by progressive dyspnea and a relentless decline in lung function, carrying a poor prognosis [1]. IPF is a rare disease with an incidence of 0.48–11.7 per 100,000 [2]. While the exact pathophysiological mechanisms underlying the development of IPF remain elusive, significant advances have been made in understanding its pathogenesis in recent years. The currently prevailing hypothesis suggests that IPF arises from repetitive alveolar epithelial injury, which leads to the activation and differentiation of fibroblasts into myofibroblasts. The excessive accumulation of these differentiated myofibroblasts and extracellular matrix components triggers aberrant tissue repair, ultimately resulting in permanent scarring and irreversible loss of pulmonary function [3]. Although the specific etiology of IPF remains unidentified, several risk factors implicated in the development of the disease have been defined. These risk factors include genetic mutations, environmental and occupational exposures, most notably cigarette smoke, viral and bacterial infections, and chronic microaspiration secondary to gastroesophageal reflux disease (GERD). Furthermore, advanced age and various alterations occurring at the cellular and molecular levels are also considered significant contributors to the pathogenesis [4,5].

Bronchoalveolar lavage (BAL) is an important diagnostic tool that is minimally invasive, well-tolerated, and capable of facilitating the diagnosis of various lung diseases. This procedure allows for the evaluation of alveolar compartments by obtaining cellular and extracellular components from the lower respiratory tract. Changes in the constituents and cellular proportions of BAL fluid reflect the underlying pathological alterations in the lung parenchyma [6]. In patients with IPF, gastric contents such as food particles, bile secretions, and pepsin have been detected in BAL fluid samples, and their presence has been associated with the progression of lung fibrosis. Furthermore, it has been reported that occult aspiration of gastric contents may play a role in the acute exacerbation of idiopathic pulmonary fibrosis in certain [7]

While some studies have indicated that medical and surgical treatment of GERD may reduce the progression of pulmonary fibrosis or lead to disease stabilization, other research suggests that antacid therapy does not significantly impact overall survival [8,9,10]. Conversely, some studies contend that antacid therapy does not improve clinical outcomes in patients with IPF and may even be associated with an increased risk of infection, particularly in those with advanced-stage disease [10]. A comprehensive systematic review and meta-analysis on this subject indicated that low-quality evidence suggests pharmacological treatment of GERD is associated with a reduction in IPF-related mortality, although no such benefit was observed for all-cause mortality; furthermore, the study emphasized an urgent need for randomized controlled trials to clarify the role of antacid therapy in IPF management [11]. Various diagnostic tests can be utilized to identify GERD, including 24 or 48 h ambulatory esophageal pH monitoring, manometric measurements showing decreased pressure in the upper and lower esophageal sphincters, and the assessment of lipid-laden alveolar macrophages (LLAMs) in respiratory secretions [12]. Previous studies have established that lipid-laden alveolar macrophages (LLAMs) serve as a marker for the microaspiration of gastric contents into the respiratory tract [13,14]. The accumulation of lipids within the cytoplasm of alveolar macrophages is evaluated using the Lipid-Laden Macrophage Index (LLMI). This index assesses both the number of lipid-laden macrophages and the relative quantity of lipids contained within them. Furthermore, the Lipid-Laden Macrophage Index is considered a potential indicator of inflammation within the respiratory tract [15].

Lipids contained within the aspirated gastric contents are phagocytosed by alveolar macrophages, and the analysis of these cells in bronchoalveolar lavage (BAL) is believed to serve as an indicator for the severity of the associated inflammatory process. The lipid-laden alveolar macrophage index (LLMI) has been found to be significantly higher in patients with suspected aspiration compared to children with other non-aspiration-related pulmonary pathologies and healthy pediatric controls. In previous ROC analyses, a cut-off value of >165 (demonstrating 98.6% sensitivity, 78.0% specificity, and 87.8% overall accuracy) was established. Consequently, an LLMI exceeding 165 has been reported as a useful diagnostic tool for the identification of aspiration [16].

T lymphocytes are derived from lymphoid progenitor cells in the bone marrow and undergo maturation in the thymus, subsequently migrating to various organs through the circulatory system. Based on the expression of specific cell surface proteins, T cells are classified into two primary subtypes: CD4+ and CD8+ cells, which recognize Major Histocompatibility Complex (MHC) class II and class I molecules, respectively. In the lung, CD4+ and CD8+ T cells function as the predominant adaptive immunocytes, participating in the clearance of pathogens and persisting thereafter as memory T cells [17].

Normally, the presence of a small amount of air within the esophagus is not considered pathological. However, increased air or distension in the esophagus on chest computed tomography (CT) may serve as an early predictive marker for connective tissue diseases, such as scleroderma, in their initial stages. Furthermore, it may be useful in diagnosing conditions where gastric acid aspiration is thought to play an etiologic role. On chest CT, the presence of air exceeding 10 mm in the coronal plane is indicative of esophageal dilation [18].

In this study, we aimed to evaluate the relationship between esophageal diameter dilated due to increased air content on chest CT and potentially associated with microaspiration and various parameters in patients with IPF, including the Lipid-Laden Macrophage Index (LLMI), differential cell counts, and the levels of BAL CD4+, BAL CD8+, and the BAL CD4+/CD8+ ratio. Furthermore, we investigated the potential impact of smoking status on these parameters and its correlation with peripheral blood CD4+ and CD8+ T-lymphocyte subsets, aiming to provide a more comprehensive understanding of the factors influencing the immunological and structural profile in IPF patients.

## 2. Materials and Methods

### 2.1. Study Population and Ethical Approval

In this retrospective study, 71 patients diagnosed with idiopathic pulmonary fibrosis (IPF) at our University Faculty of Medicine, Department of Chest Diseases, between 2016 and 2023 were evaluated. The diagnosis of IPF was established by a multidisciplinary interstitial lung disease committee comprising specialists in pulmonology, radiology, pathology, thoracic surgery, and rheumatology. Initially, 92 patients were identified; however, 9 were excluded due to severe lung function limitations and hypoxemia precluding bronchoscopy. Of the remaining 83 patients who underwent fiberoptic bronchoscopy, 12 were excluded because their bronchoalveolar lavage (BAL) fluid samples contained >5% epithelial cells, indicating oropharyngeal contamination. Ultimately, 71 patients with complete data for BAL differential cell counts, BAL and peripheral blood T-lymphocyte subsets (CD4+, CD8+), and Lipid-Laden Macrophage Index (LLMI) were included. The study was conducted in accordance with the Declaration of Helsinki and approved by the local Ethics Committee (Decision No: 2023/4616).

### 2.2. Fiberoptic Bronchoscopy and BAL Procedure

The procedure was performed under sedation and local anesthesia (midazolam and lidocaine) following standard clinical protocols. BAL fluid was obtained from the segment with predominant parenchymal involvement or from the right middle lobe in cases of diffuse involvement. A total of 100 mL of 0.9% saline (in 20 mL aliquots) was instilled through the bronchoscope and immediately recovered by gentle aspiration. Samples were promptly transported to the cytology unit for differential cell counting and LLMI analysis. Simultaneous BAL and peripheral blood samples were sent to the immunology laboratory for CD4+ and CD8+ T-lymphocyte quantification via flow cytometry using a BD FACSCanto II (BD Biosciences, San Jose, CA, USA).

### 2.3. Lipid-Laden Macrophage Index (LLMI) Assessment

Alveolar macrophage lipid content was identified using Oil Red O staining (Sigma-Aldrich, St. Louis, MO, USA). According to the protocol described by Colombo and Hallberg, intracellular lipid accumulation was graded in 100 consecutive alveolar macrophages on a scale of 0 to 4 based on opacification (0: no opacification; 1: up to 1/4 opaque; 2: 1/4 to 1/2 opaque; 3: 1/2 to 3/4 opaque; 4: completely opaque) [13]. The final LLMI was calculated by summing these scores, yielding a total range of 0 to 400.

### 2.4. Radiological Evaluation of Esophageal Parameters

Chest computed tomography (CT) scans were performed using a Somatom Drive (Siemens Healthineers, Erlangen, Germany) scanner. The images were analyzed using a standard mediastinal window (width 400 HU, level 40 HU). The esophageal diameter was measured in the axial plane at four distinct levels: the mid-aortic arch (L1), the main carina (L2), the superior pulmonary vein (L3), and 1 cm above the diaphragmatic hiatus (L4) [19]. Consistent with previous literature and established radiological criteria for systemic sclerosis-related interstitial lung disease, an esophageal diameter exceeding 10 mm was defined as esophageal dilation [18]. Furthermore, the maximum coronal diameter from the thoracic inlet to the esophageal hiatus was recorded [20]. A hiatal hernia was diagnosed if the esophageal hiatus was >1.5 cm and/or if gastric structures were visualized above the hiatus [21].

### 2.5. AI Assistance

During the preparation of this manuscript, Gemini 3 Flash (Google, Mountain View, CA, USA) was utilized for English language translation, grammatical refinement, and enhancing technical readability. The authors have reviewed and edited the output to ensure scientific accuracy and take full responsibility for the final content.

### 2.6. Statistical Analysis

Statistical analysis was performed using SPSS for Windows, version 28.0 (SPSS Inc., Chicago, IL, USA). Data distribution was assessed via histograms, coefficients of variation, and skewness-kurtosis coefficients. Intergroup comparisons were conducted using the Mann–Whitney U test. Correlations between variables were evaluated using Pearson’s correlation coefficient. A *p*-value of <0.05 was considered statistically significant. Due to the sample size and exploratory nature of this study, multivariable analysis for confounders was not performed.

Declaration of Generative AI and AI-Assisted Technologies in the Writing Process: During the preparation of this manuscript, the authors used Gemini 3 Flash exclusively for English language translation and to enhance the technical readability of the text. This tool did not assist in data collection, statistical analysis, or the interpretation of the clinical results. Following its use, all machine-generated content was thoroughly reviewed, verified, and edited by the authors. The authors assume full responsibility for the content and integrity of the final published article.

## 3. Results

### 3.1. Baseline Characteristics and Smoking Status

The mean age of the 71 patients included in the study was 68.09 ± 13.25 years. The majority of the study population was male (69%, *n* = 49), while females accounted for 31% (*n* = 22). Regarding smoking habits, 56.3% of the patients (*n* = 40) were active or former smokers. Gender-specific analysis revealed a significant disparity in smoking history: 79.6% (*n* = 39) of male patients had a smoking history, whereas 95.5% (*n* = 21) of female patients were non-smokers. Detailed demographic and clinical characteristics are summarized in Table 1.

### 3.2. BAL Fluid Cytology, LLMI, and T-Lymphocyte Subsets

The cytological analysis of BAL fluid showed a predominant macrophage count (71.37% ± 19.98%), followed by neutrophils (12.99% ± 18.02%), lymphocytes (12.13% ± 9.62%), epithelial cells (3.68% ± 1.38%), and eosinophils (0.44% ± 1.25%).

Intracellular lipid accumulation was detected in the macrophages of 63.38% (*n* = 45) of the 71 patients. Among these patients, the average macrophage lipid load was 15.8%. The mean Lipid-Laden Macrophage Index (LLMI) for the study population was determined to be 58.29 out of a maximum score of 400 (Table 2).

Immunological analysis of BAL fluid showed mean CD4+ and CD8+ T-lymphocyte percentages of 38.18% ± 19.72% and 34.23% ± 21.40%, respectively, with a mean BAL CD4+/CD8+ ratio of 1.69 ± 1.67. Peripheral blood analysis revealed similar T-cell distributions, with mean CD4+ and CD8+ levels of 36.28% ± 10.14% and 31.01% ± 11.02%, respectively.

### 3.3. Impact of Smoking Status on BAL and Immunological Parameters

The comparative analysis of patients based on smoking status is presented in Table 3. There were no statistically significant differences between smokers and non-smokers regarding BAL differential cell counts, including macrophages, neutrophils, and lymphocytes (*p* > 0.05). Similarly, neither the percentage of lipid-laden macrophages nor the total Lipid-Laden Macrophage Index (LLMI) showed a significant disparity between the two groups (*p* = 0.17).

However, a statistically significant difference was observed in BAL CD4+ T-lymphocyte levels. Patients who did not smoke exhibited significantly higher BAL CD4+ values compared to smokers (43.58 ± 19.49 vs. 34.00 ± 19.10, respectively; *p* = 0.042). No significant differences were found in BAL CD8+, BAL CD4+/CD8+ ratio, or peripheral blood T-lymphocyte subsets between the groups (*p* > 0.05).

### 3.4. Radiological Assessment of Esophageal Diameters

The esophageal diameters measured at four distinct anatomical levels on chest CT are summarized in Table 4. The mean esophageal diameter increased progressively from the proximal to the distal segments. The highest mean diameter was observed at the L4 level (1 cm above the diaphragmatic hiatus), measured at 10.96 ± 6.44 mm (range: 1.2–30 mm). The overall mean esophageal diameter across all levels was 6.06 ± 4.88 mm. When analyzed by gender, the mean diameter was slightly higher in male patients compared to females (6.46 ± 4.85 mm vs. 5.15 ± 4.91 mm, respectively).

### 3.5. Esophageal Dilation and Hiatal Hernia Prevalence

The maximum esophageal diameter measured at any level reached up to 30 mm, with a mean maximum diameter across all levels of 11.09 ± 6.64 mm. Esophageal dilation, defined as a diameter exceeding 10 mm at any of the measured levels, was present in 52.1% (n = 37) of the study population. Additionally, a hiatal hernia identified by an esophageal hiatus > 1.5 cm and/or the presence of gastric structures above the hiatus was detected in 36.6% (n = 26) of the patients.

### 3.6. Correlation Analysis Between Esophageal Parameters and BAL Findings

The correlation analysis between esophageal measurements and immunological/cytological parameters is summarized in Table 5. No statistically significant correlation was found between esophageal diameters (at any level), mean maximum diameter, or hiatal hernia and the percentage of lipid-laden macrophages or the total LLMI (*p* > 0.05).

However, a statistically significant positive correlation was identified between the presence of hiatal hernia and BAL neutrophil counts (r = 0.298, *p* = 0.012), indicating a weak-to-moderate association where the presence of hiatal hernia corresponds with increased neutrophilic inflammation.

Notably, a significant negative correlation was observed between the esophageal diameter at the L4 level and BAL CD4+ T-lymphocyte levels (r = −0.267, *p* = 0.024). Similarly, the mean maximum esophageal diameter was negatively correlated with BAL CD4+ counts (r = −0.288, *p* = 0.015). Additionally, peripheral blood CD4+ levels showed a positive correlation with the esophageal diameter at the L2 level (r = 0.276, *p* = 0.020).

### 3.7. Comparison of Neutrophil Counts Based on Hiatal Hernia Status

A comparative analysis was performed to evaluate the impact of hiatal hernia on pulmonary inflammation. Patients with hiatal hernia exhibited significantly higher BAL neutrophil percentages compared to those without hiatal hernia (20.0% ± 4.39% vs. 8.93% ± 2.0%, respectively; *p* = 0.047). The neutrophil counts in the hiatal hernia group reached as high as 70%, suggesting a more pronounced inflammatory response in these individuals (Table 6).

### 3.8. Impact of Smoking on BAL CD4+ T-Lymphocyte Levels

A correlation analysis revealed a statistically significant, weak-to-moderate negative correlation between smoking status and BAL CD4+ T-lymphocyte levels. To further investigate this relationship, patients were stratified by their smoking history. Non-smokers (n = 31) had significantly higher mean BAL CD4+ levels compared to smokers (n = 40) (43.58 ± 19.49% vs. 34.00 ± 19.10%, respectively; *p* = 0.042), as shown in Table 7.

### 3.9. Correlation Analysis in the Esophageal Dilation Subgroup

In the subgroup of patients with esophageal dilation (diameter > 10 mm, n = 37), no statistically significant correlation was found between esophageal diameters at any level and lipid-laden macrophage percentage or LLMI (*p* > 0.05). The detailed correlation coefficients for this subgroup are presented in Table 8.

### 3.10. Interaction Between Smoking, Esophageal Dilation, and Lipid Parameters

A further stratified analysis was conducted on patients presenting with both esophageal dilation (>10 mm) and a significant lipid load (LLMI > 100/400). Within this specific cohort (n = 37), the population was divided based on smoking history: smokers (*n* = 21) and non-smokers (n = 16). Correlation analysis showed no statistically significant relationship between esophageal diameters at any level (L1–L4) and lipid parameters (LLM% or LLMI) in either group (*p* > 0.05). These findings suggest that smoking status does not significantly modulate the relationship between structural esophageal changes and markers of microaspiration in this subgroup.

## 4. Discussion

The results of our study suggest that structural esophageal abnormalities, specifically distal esophageal dilation and hiatal hernia, are significantly associated with altered immunological and inflammatory profiles in patients with idiopathic pulmonary fibrosis (IPF). Our most striking finding was the significant negative correlation between the esophageal diameter at the diaphragmatic level (L4) and bronchoalveolar lavage (BAL) CD4+ T-lymphocyte counts. Furthermore, the presence of hiatal hernia was associated with a more than twofold increase in BAL neutrophil percentages, suggesting that anatomical defects facilitating microaspiration may drive neutrophilic inflammation and modulate local cellular immunity in the fibrotic lung.

Idiopathic pulmonary fibrosis is a devastating disease with a prognosis often compared to malignant lung tumors [1]. While its etiology is multifactorial, gastroesophageal reflux disease (GERD) and subsequent chronic microaspiration have been identified as key contributors to disease progression and acute exacerbations [5,22]. Current literature suggests that the repeated inhalation of gastric contents including acid, pepsin, and bile salts induces repetitive alveolar epithelial injury, a hallmark of IPF pathogenesis [6]. Our findings support this “microaspiration hypothesis” by showing that structural markers of GERD (esophageal dilation and hiatal hernia) correlate with increased airway inflammation.

The radiological assessment of the esophagus serves as a non-invasive surrogate marker for esophageal dysfunction. We found that over half of our patients (52.1%) had an esophageal diameter exceeding 10 mm, and 36.6% presented with hiatal hernia. These rates are consistent with previous reports indicating a high prevalence of GERD-related features in IPF cohorts [18]. However, we specifically noted that the L4 level (distal esophagus) was the most dilated segment (10.96 ± 6.44 mm). The significant negative correlation between L4 diameter and BAL CD4+ levels (r = −0.267, *p* = 0.024)is a novel observation. While CD4+ T-cells are essential for immune homeostasis, their reduction in the presence of significant esophageal dilation may indicate a localized immune exhaustion or shift in the lung microenvironment caused by chronic aspiration-induced injury.

Another critical finding was the relationship between hiatal hernia and neutrophilic alveolitis. Patients with hiatal hernia exhibited a mean neutrophil count of 20.0%, significantly higher than the 8.93% observed in those without hernia (*p* = 0.047). Neutrophils are known to release various proteases and reactive oxygen species that promote fibroproliferation. The presence of a hiatal hernia likely compromises the anti-reflux barrier more severely than transient reflux, leading to a higher volume of aspiration and a sustained neutrophilic response. This aligns with the work of Richardson et al., who suggested that hiatal hernia is a predictor of more severe pulmonary involvement in connective tissue diseases [23].

The findings of our study suggest a potential complex interaction between structural esophageal alterations and lifestyle factors, such as smoking, on the pulmonary immune microenvironment. Our data indicated that both smoking and distal esophageal dilation (L4 level) may independently be associated with a decrease in bronchoalveolar lavage (BAL) CD4+ T-lymphocyte levels. Specifically, smokers in our cohort exhibited lower CD4+ counts compared to non-smokers (34.00 ± 19.10% vs. 43.58 ± 19.49%, *p* = 0.042). When considered alongside the observed significant negative correlation between distal esophageal diameter and CD4+ levels (r = −0.267, *p* = 0.024), these results point toward a cumulative suppressive impact on local immune cells. This interplay suggests that IPF patients with comorbid esophageal dysfunction and a history of smoking may represent a high-risk clinical phenotype with a more profoundly compromised immune profile.

The cellular profile of bronchoalveolar lavage (BAL) fluid in healthy, non-smoking individuals typically consists of 80–90% alveolar macrophages, 5–15% lymphocytes, 1–3% neutrophils, and less than 1% eosinophils or mast cells. In healthy smokers, while the total cell count significantly increases due to macrophage recruitment, the percentages of other cells often remain stable, though the CD4+/CD8+ ratio may fluctuate. In patients with idiopathic pulmonary fibrosis (IPF), however, the BAL profile is often non-specific but typically characterized by macrophage dominance alongside moderate increases in neutrophils and eosinophils. Our cohort reflected this trend, with mean percentages of 71.37% for macrophages, 12.13% for lymphocytes, 12.99% for neutrophils, and 0.44% for eosinophils.

The clinical significance of BAL neutrophilia in IPF remains a subject of ongoing debate. While some studies suggest no significant correlation with survival [24], others, such as Kinder et al., have identified elevated neutrophil percentages as an independent predictor of early mortality [25]. Recent evidence also highlights the role of granulocyte colony-stimulating factor (G-CSF) in driving this neutrophilic response, with higher concentrations correlating with reduced survival rates [26]. Neutrophils contribute to the fibrotic process by releasing neutrophil elastase, which promotes extracellular matrix remodeling and fibroblast differentiation into myofibroblasts [27,28]. Consistent with these pathogenetic mechanisms, our study confirmed an increased neutrophil ratio in IPF patients, particularly in those with comorbid hiatal hernia (*p* = 0.012).

The interaction between the adaptive immune system and IPF is further complicated by the aging process. IPF predominantly affects older adults, a population characterized by immunosenescence the gradual decline of the immune system. The involution of the thymus reduces the production of naive T-cells, which are often replaced by memory T-cells and regulatory T-cells in the aging lung [29,30,31]. While some studies have found no significant difference in BAL CD4+/CD8+ ratios between smokers and non-smokers among IPF patients [24,32], our study observed a notable distinction. Non-smokers in our cohort had significantly higher BAL CD4+ levels (43.58%) compared to smokers (34.00%, *p* = 0.042). Given that Th1 cells (a subset of CD4+) play a potentially anti-fibrotic role by producing IFN-gamma which inhibits collagen synthesis the reduction in CD4+ cells in smokers may provide a different perspective on how smoking exacerbates IPF pathogenesis [33].

Furthermore, we identified a significant positive correlation between the esophageal diameter at the L2 level and peripheral blood CD4+ counts (r = 0.276, *p* = 0.020). Previous studies have suggested that circulating naive CD4+ levels may correlate with baseline forced vital capacity (FVC) and that their decline might track with disease progression [34]. While the exact mechanism behind the L2-Blood CD4+ link requires further elucidation, our findings suggest that radiological esophageal parameters could serve as potential surrogate markers for immune status or disease severity. These observations provide a preliminary framework for larger, prospective studies to evaluate whether esophageal measurements can aid in predicting clinical outcomes or guiding individualized therapeutic strategies in IPF.

IPF is characterized by a higher prevalence in older male adults, with several risk factors identified in its pathogenesis, including cigarette smoke, environmental or occupational exposures, chronic microaspiration due to gastroesophageal reflux disease (GERD), and aging [4,5]. The demographic profile of our cohort, with a mean age of 68.09 ±13.25 years and a male predominance (69%), is consistent with global epidemiological data. Furthermore, 56.3% of our patients were smokers, reinforcing the significance of this risk factor in the IPF population.

While the precise etiology of IPF remains elusive, the prevalence of GERD in this population is notably high, with some studies reporting rates as high as 62.3% [12,22]. Interestingly, GERD in IPF patients is frequently asymptomatic; typical symptoms such as heartburn, regurgitation, and dysphagia are absent in a significant majority of cases [35]. Consequently, clinical symptoms alone are insufficient screening tools for GERD in IPF. In this context, radiological assessment of the esophagus via CT provides a valuable non-invasive alternative. Bhalla et al. [18] demonstrated that an esophageal diameter exceeding 10 mm is a reliable indicator of dilation, reporting asymptomatic dilation in 80% of patients with systemic sclerosis-related interstitial lung disease. They suggested that early detection of esophageal involvement is crucial for managing complications related to dysmotility and chronic reflux.

In our study, the mean esophageal diameter across all levels was 11.09 ± 6.64 mm, with 52.1% (*n* = 37) of patients exhibiting dilation (diameter > 10 mm) at least at one level. Additionally, hiatal hernia defined as an esophageal hiatus > 1.5 cm or the presence of gastric structures above the hiatus was identified in 36.6% of our patients (*n* = 26). These findings further underscore the high prevalence of structural esophageal abnormalities in IPF, even in the absence of overt clinical symptoms.

The presence of lipid-laden macrophages (LLM) in BAL fluid is a recognized marker of microaspiration, though its diagnostic utility remains a subject of intense debate [28,36]. Lipid accumulation can occur through endogenous pathways (e.g., breakdown of distal cell membranes in obstructive lesions) or exogenous pathways (e.g., aspiration of gastric contents or inhalation of lipids) [27]. While various cut-off values for the Lipid-Laden Macrophage Index (LLMI) have been proposed in the literature ranging from 85/400 to 165/400 [13,16,24] these studies were predominantly conducted in pediatric populations. Our study found a mean LLMI of 58.29/400, which is lower than the previously reported cut-offs. This discrepancy may be attributed to age-related physiological differences, as data regarding LLMI in adult IPF patients remain limited. Contrary to our findings in adult IPF patients, studies in pediatric populations have demonstrated that the presence of lipid-laden macrophages in BAL fluid is a valuable tool for diagnosing GERD and chronic cough. For instance, Pekcan et al. highlighted that identifying lipid-laden macrophages could lead to a successful diagnosis and treatment of GERD even in cases where initial findings were inconclusive. However, the absence of a significant correlation between esophageal diameter and LLM in our cohort supports the hypothesis that the diagnostic utility of LLM may differ significantly between pediatric populations and adult patients with interstitial lung diseases, where chronic structural changes might be more indicative of disease dynamics than cellular lipid markers [37].

The relationship between GERD and IPF appears to be bidirectional. Increased intrathoracic pressure in IPF, combined with factors like smoking and advanced age, may predispose patients to GERD. Conversely, chronic microaspiration of gastric contents can induce persistent alveolar inflammation, potentially accelerating the progression of pulmonary fibrosis [29,38,39]. Although the benefit of antacid therapy in IPF remains controversial with some studies suggesting improved survival [9] and others warning of increased infection risks in advanced disease [10] current guidelines suggest that management should be individualized, particularly for patients with symptomatic GERD [1]. Our findings, consistent with those of Roth et al., suggest that structural esophageal dysfunction and dilation are linked to more severe disease profiles and impaired pulmonary function [40,41].

To our knowledge, there is a scarcity of studies in the literature specifically investigating the relationship between radiological esophageal measurements and BAL microaspiration markers in IPF patients. Based on the hypothesis that a dilated esophagus facilitates gastroesophageal reflux (GERD) and subsequent microaspiration thereby influencing the pathogenesis or quality of life in IPF we investigated the correlation between HRCT-derived esophageal diameters and the Lipid-Laden Macrophage Index (LLMI), a biological indicator of aspiration.

Our analysis revealed no statistically significant correlation between the percentage of lipid-laden macrophages and esophageal diameters at any level (L1, L2, L3, L4; *p* = 0.234, 0.167, 0.335, 0.203, respectively), the overall mean diameter (*p* = 0.178), or the presence of hiatal hernia (*p* = 0.333). Similarly, the LLMI did not show significant associations with esophageal measurements or hiatal hernia (*p* > 0.05) (Table 5).

These findings align with a recent systematic review by Lawlor et al., which evaluated five studies involving 720 patients to determine if LLMI could serve as a reliable marker for GERD in BAL fluid. Despite various proposed cut-off values (ranging from 85 to 165), the review concluded that the current literature does not support LLMI as a highly sensitive or specific marker for aspiration, emphasizing the need for further research to establish its clinical utility [42].

In our cohort, a subgroup of 37 patients exhibited both esophageal dilation (diameter > 10 mm) and a significant lipid load (LLMI > 100/400). Even within this specific subgroup, no significant correlation was found between structural esophageal changes and lipid markers, further supporting the conclusions of Lawlor et al. (Table 8) [42]. When the current literature and our results are considered together, it is evident that LLMI cannot independently confirm or exclude microaspiration. However, when combined with other clinical observations and diagnostic tests, an elevated LLMI score may still increase the clinical suspicion of “silent aspiration.

Furthermore, we stratified the patients with significant esophageal dilation and high LLMI by their smoking status (21 smokers vs. 16 non-smokers). In both subgroups, separate correlation analyses between esophageal diameter and LLMI yielded no significant results (Table 9). This suggests that smoking status does not significantly alter the relationship or lack thereof between structural esophageal changes and lipid-laden macrophage counts in our study population.

### Limitations

Our study has several limitations that should be considered when interpreting the results. The most significant limitation is the lack of a healthy control group. Due to the invasive nature of bronchoscopy and bronchoalveolar lavage (BAL), as well as the inherent ethical and practical challenges in recruiting healthy volunteers for such procedures, a control group could not be established. Consequently, our findings on BAL cellular profiles and their relationship with esophageal diameter and smoking remain descriptive within the context of IPF.

Another limitation is the relatively small sample size (n = 71). While our cohort provided sufficient data to identify significant correlations, a larger and more diverse study population would enhance the generalizability of our findings. Increased statistical power would allow for a more robust investigation into how CD4+ T-lymphocyte counts and neutrophils interact with esophageal structural changes and smoking history in the pathogenesis and prognosis of IPF.

Furthermore, we utilized radiological esophageal measurements as surrogate markers for GERD rather than the ‘gold standard’ 24 h pH-impedance monitoring or esophageal manometry. While HRCT provides valuable structural insights into esophageal dilation, it cannot evaluate the actual reflux frequency or the acidity of the aspirated content. Consequently, the lack of functional testing limits our ability to draw direct causal inferences between the severity of reflux and the observed immunological changes. Despite these constraints, our results demonstrated correlations similar to the known effects of smoking, suggesting that structural esophageal changes—independent of the Lipid-Laden Macrophage Index (LLMI) represent a factor that should not be overlooked in the management of IPF. These findings serve as a preliminary framework, highlighting the need for larger, prospective, and multi-center studies to further elucidate the causal pathways involved.

Due to the sample size and exploratory nature of this study, multivariable analysis for confounders was not performed.

## 5. Conclusions

In conclusion, our study suggests that structural esophageal abnormalities, such as distal esophageal dilation and hiatal hernia, are significantly associated with altered local immune profiles in patients with IPF. The observed negative correlation between distal esophageal diameter (L4) and BAL CD4+ T-lymphocyte counts, along with the association between hiatal hernia and increased neutrophilic inflammation, points toward a potential interplay between esophageal dysfunction and the pulmonary immune microenvironment.

While these findings underscore the potential of radiological esophageal assessment via HRCT as a non-invasive tool to identify high-risk clinical phenotypes, they should be interpreted as preliminary. Our results indicate that structural changes, independent of the LLMI, represent a factor that should be integrated into the clinical monitoring of IPF. However, due to the lack of functional esophageal testing and the exploratory nature of this study, further large-scale prospective and multi-center research is warranted to establish definitive causal relationships and determine if managing esophageal dysfunction can improve long-term clinical outcomes.

## Figures and Tables

**Table 1:** Demographic Characteristics of Patients with IPF.

Characteristic (*n* = 71)	Mean ± SD/*n* (%)
Age (years)	68.09 ± 13.25
Gender	
Female	22 (31%)
Male	49 (69%)
Smoking Status	
Non-smoker	31 (42.2%)
Smoker	40 (56.3%)

**Table 2:** BAL differential cell counts, LLMI, and T-lymphocyte subsets in patients with IPF.

Parameters	Mean ± SD	Range (Min–Max)
BAL Cytology (%) (*n* = 71)		
Macrophage	71.37 ± 19.98	15–93
Epithelial Cell	3.68 ± 3.38	1–5
Eosinophil	0.44 ± 1.25	0–5
Neutrophil	12.99 ± 18.02	0–70
Lymphocyte	12.13 ± 9.62	0–55
Lipid Parameters (*n* = 45)		
Lipid-Laden Macrophage (%)	15.80 ± 20.47	0–80
LLMI (0–400)	58.29 ± 77.96	0–300
T-Lymphocyte Subsets		
BAL CD4+ (%) (*n* = 71)	38.18 ± 19.72	0–80
BAL CD8+ (%) (*n* = 71)	34.23 ± 21.40	0–85
BAL CD4+/CD8+ ratio (*n* = 69)	1.69 ± 1.67	0–7.75
Blood CD4+ (%) (*n* = 71)	36.28 ± 10.14	5–61
Blood CD8+ (%) (*n* = 71)	31.01 ± 11.02	4–62

LLMI, Lipid-Laden Macrophage Index; BAL, Bronchoalveolar Lavage; CD, Cluster of Differentiation.

**Table 3:** Comparison of BAL and Blood Parameters Based on Smoking Status.

Parameters	Non-Smokers (*n* = 31) Mean ± SD	Smokers (*n* = 40) Mean ± SD	*p*-Value
Macrophage (%)	67.19 ± 20.33	71.05 ± 19.80	0.424
Epithelial Cell (%)	5.71 ± 3.34	5.65 ± 3.45	0.942
Eosinophil (%)	0.35 ± 1.08	0.50 ± 1.45	0.643
Neutrophil (%)	13.74 ± 18.38	12.40 ± 17.95	0.758
Lymphocyte (%)	14.03 ± 10.74	10.65 ± 8.49	0.143
Lipid-Laden Macrophage (%)	19.39 ± 24.38	13.03 ± 16.60	0.210
BAL CD4+ (%)	43.58 ± 19.49	34.00 ± 19.10	0.042 *
BAL CD8+ (%)	33.61 ± 18.33	34.70 ± 23.73	0.834
BAL CD4+/CD8+ Ratio	1.91 ± 1.78	1.52 ± 1.59	0.347
Blood CD4+ (%)	37.90 ± 11.54	35.03 ± 8.85	0.238
Blood CD8+ (%)	28.81 ± 11.67	32.73 ± 10.31	0.139
LLMI (0–400)	73.16 ± 92.00	46.77 ± 63.92	0.170

* Statistically significant (*p* < 0.05). LLMI, Lipid-Laden Macrophage Index; BAL, Bronchoalveolar Lavage.

**Table 4:** Esophageal Diameter Measurements on Chest CT (*n* = 71).

Measurement Level	Mean ± SD (mm)	Range (Min–Max)
L1 (Mid-aortic arch level)	3.21 ± 4.37	0–20.1
L2 (Main carina level)	4.70 ± 5.90	0–23.7
L3 (Superior pulmonary vein level)	5.35 ± 6.87	0–29.36
L4 (1 cm above diaphragmatic hiatus)	10.96 ± 6.44	1.2–30.0
Overall Mean Diameter	6.06 ± 4.88	0.30–22.45
Overall Mean Diameter (Male)	6.46 ± 4.85	0.40–22.45
Overall Mean Diameter (Female)	5.15 ± 4.91	0.30–21.33

**Table 5:** Comprehensive Correlation Matrix of Esophageal, Clinical, and BAL Parameters (*n* = 71).

Correlations N:71		L1	L2	L3	L4	Max Mean	Hiatal Hernia	Age	Gender	Smoking
Macrophage %	r	0.020	−0.114	−0.098	−0.164	−0.141	−0.217	0.107	0.045	0.096
	*p*	0.866	0.342	0.417	0.171	0.241	0.069	0.375	0.712	0.424
Epithelial Cell %	r	−0.226	−0.124	−0.068	−0.067	−0.082	0.021	0.028	0.008	−0.009
	*p*	0.058	0.303	0.572	0.581	0.494	0.861	0.814	0.948	0.942
Eosinophil %	r	0.014	0.032	−0.131	0.040	0.031	−0.053	0.040	0.085	0.056
	*p*	0.910	0.792	0.275	0.742	0.795	0.658	0.743	0.479	0.643
Neutrophil %	r	0.042	0.173	0.086	0.187	0.171	0.298 *	0.007	0.006	−0.037
	*p*	0.726	0.148	0.477	0.119	0.153	0.012	0.951	0.959	0.758
Lymphocyte %	r	0.019	0.000	0.091	0.045	0.030	−0.056	−0.231	−0.147	−0.176
	*p*	0.875	0.999	0.449	0.709	0.804	0.643	0.052	0.220	0.143
LLM %	r	−0.143	−0.166	−0.116	−0.153	−0.162	0.117	−0.202	−0.186	−0.155
	*p*	0.234	0.167	0.335	0.203	0.178	0.333	0.091	0.120	0.196
LLMI	r	−0.158	−0.176	−0.100	−0.146	−0.155	0.112	−0.201	−0.207	−0.169
	*p*	0.189	0.142	0.408	0.224	0.197	0.354	0.092	0.084	0.159
BAL CD4+	r	−0.197	−0.195	−0.216	−0.267 *	−0.288 *	−0.171	0.071	−0.350	−0.243 *
	*p*	0.100	0.103	0.070	0.024	0.015	0.153	0.554	0.003	0.042
BAL CD8+	r	0.001	−0.043	−0.064	0.021	0.002	−0.029	0.011	0.000	0.025
	*p*	0.996	0.721	0.596	0.859	0.985	0.812	0.926	1.000	0.834
BAL CD4+/CD8+	r	−0.097	−0.157	−0.198	−0.226	−0.235	−0.108	0.015	−0.158	−0.115
	*p*	0.428	0.198	0.103	0.062	0.052	0.375	0.903	0.194	0.347
Kan CD4+	r	0.213	0.276 *	0.076	0.094	0.111	0.106	−0.142	−0.111	−0.142
	*p*	0.075	0.020	0.526	0.434	0.356	0.377	0.239	0.355	0.238
Kan CD8+	r	−0.039	−0.126	0.001	−0.002	0.001	0.007	−0.089	0.154	0.178
	*p*	0.750	0.294	0.994	0.988	0.992	0.954	0.461	0.200	0.139

* *p* < 0.05. r: Pearson correlation coefficient. LLM: Lipid-laden macrophages. LLMI: Lipid-Laden Macrophage Index. Max Mean: Mean of maximum esophageal diameters.

**Table 6:** Comparison of Mean BAL Neutrophil Values in IPF Patients with and without Hiatal Hernia.

Hiatal Hernia Status	Neutrophil Mean ± SD (%)	Neutrophil Range (Min–Max)	*p*-Value
Present (*n* = 26)	20.0 ± 4.39	1–70	0.047 *
Absent (*n* = 45)	8.93 ± 2.0	0–58	

* Statistically significant (*p* < 0.05).

**Table 7:** Comparison of Mean BAL CD4+ Values Between Smoker and Non-Smoker IPF Patients.

Smoking Status	BAL CD4+ Mean ± SD (%)	BAL CD4+ Range (Min–Max)	*p*-Value
Non-Smoker (*n* = 31)	43.58 ± 19.49	6–80	0.042 *
Smoker (*n* = 40)	34.00 ± 19.10	0–74	

* Statistically significant (*p* < 0.05).

**Table 8:** Relationship Between Esophageal Diameters and Lipid Parameters in Patients with Esophageal Dilation (>10 mm).

Esophageal Parameters (*n* = 37)		Lipid-Laden Macrophage (%)	Lipid-Laden Macrophage Index
L1	r	−0.222	−0.261
	*p*	0.186	0.119
L2	r	−0.179	−0.191
	*p*	0.288	0.257
L3	r	0.000	−0.007
	*p*	0.999	0.968
L4	r	0.000	−0.058
	*p*	0.998	0.734
Overall Mean Diameter	r	−0.123	−0.155
	*p*	0.468	0.358

**Table 9:** Correlations between Esophageal Diameters and Lipid Parameters Stratified by Smoking Status (Subgroup with Diameter > 10 mm and LLMI > 100/400).

Esophageal Parameters		Smokers (*n* = 21)		Non-Smokers (*n* = 16)	
		LLM (%)	LLMI	LLM (%)	LLMI
L1	r	−0.224	−0.251	−0.244	−0.310
	*p*	0.330	0.273	0.362	0.242
L2	r	−0.215	−0.247	−0.099	−0.090
	*p*	0.350	0.281	0.717	0.741
L3	r	−0.190	−0.217	0.166	0.157
	*p*	0.410	0.345	0.538	0.562
L4	r	0.170	0.082	−0.269	−0.278
	*p*	0.462	0.725	0.314	0.297
Overall Mean Diameter	r	−0.160	−0.210	−0.104	−0.126
	*p*	0.487	0.360	0.701	0.643

## Data Availability

The datasets analyzed during the current study are not publicly available due to patient privacy and institutional data protection policies but are available from the corresponding author (Celalettin Korkmaz) on reasonable request.

## Abbreviations

IPF	Idiopathic Pulmonary Fibrosis
BAL	Bronchoalveolar Lavage
LLMI	Lipid-Laden Macrophage Index
GERD	Gastroesophageal Reflux Disease
CT	Computed Tomography
HRCT	High-Resolution Computed Tomography
PFT	Pulmonary Function Test
DLCO	Diffusing Capacity of the Lungs for Carbon Monoxide
6MWT	6-Minute Walk Test
ED	Esophageal Diameter
BMI	Body Mass Index
SD	Standard Deviation
NS	Non-smoker
S	Smoker
Ex-S	Ex-smoker
